# The Brief Case: Cutaneous ulceration associated with acalabrutinib treatment

**DOI:** 10.1128/jcm.01583-24

**Published:** 2025-03-12

**Authors:** Victor Luzarraga, Céline Nourrisson, Florence Anglade, Carole Chevenet, Philippe Poirier, Maxime Moniot

**Affiliations:** 1 Service de Parasitologie-Mycologie, CHU Clermont-Ferrand, 3IHP, Clermont-Ferrand, France; 2 Microbes, Intestin, Inflammation et Susceptibilité de l'Hôte (M2iSH), UMR Inserm/Université Clermont Auvergne U1071, USC INRAE 27006 , Clermont-Ferrand, France; 3 Service de Maladies Infectieuses et Tropicales, CHU Clermont-Ferrand 55174 , Clermont-Ferrand, France; 4 Service d’Anatomie et Cytologie Pathologiques, CHU Clermont-Ferrand 55174 , Clermont-Ferrand, France; Mayo Clinic Minnesota, Rochester, Minnesota, USA

**Keywords:** cutaneous alternariosis, acalabrutinib, chronic lymphocytic leukemia, *Alternaria alternata *species complex, invasive fungal infection, phaeohyphomycosis, bruton tyrosine kinase, BTK inhibitors

## CASE

**Fig 1:**
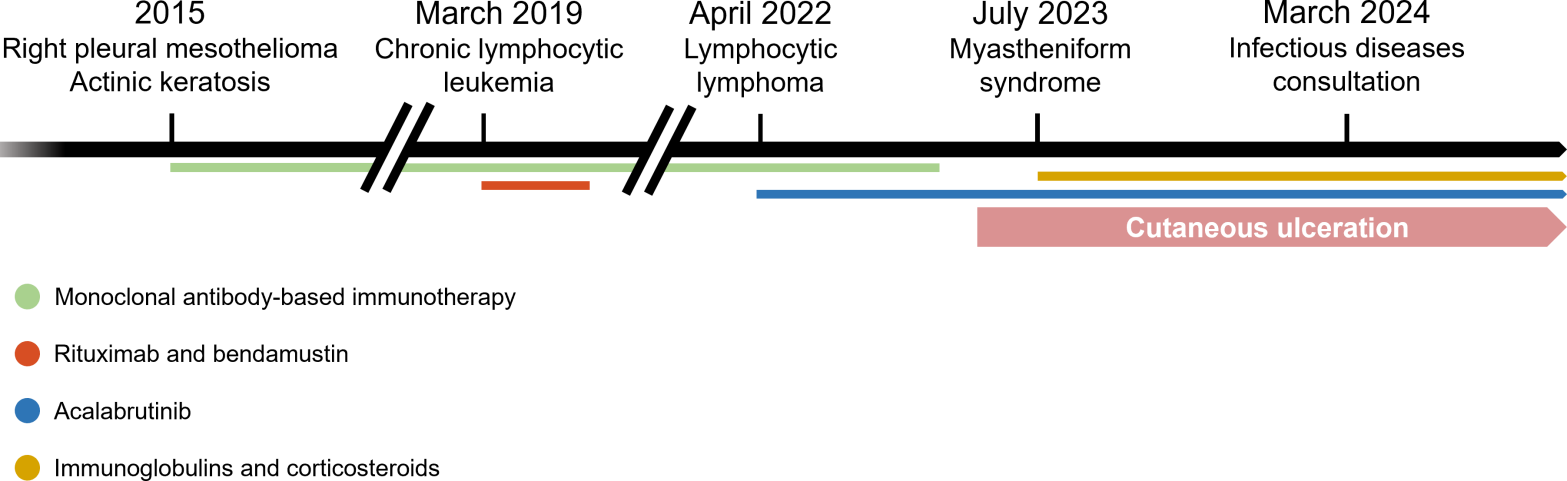
Patient history timeline. The figure summarizes the key clinical facts and associated treatments.

A 72-year-old man was admitted to the infectious diseases department on March 2024 for a cutaneous ulceration on the right thigh. The patient had a history of actinic keratosis and right pleural mesothelioma diagnosed in 2015, whose last line of treatment initiated in December 2022 was ipilimumab and nivolumab (Fig. 1). Moreover, a chronic lymphocytic leukemia (CLL) diagnosed on March 2019 was treated with rituximab and bendamustin for 6 months following initial diagnosis. A progression of the CLL to lymphocytic lymphoma was diagnosed in April 2022 and treated with acalabrutinib with the improvement of hematological disease. At the time of admission, the patient was treated with acalabrutinib (100 mg per day), in combination with IV immunoglobulins (Privigen) and corticosteroids (60 mg per day) since July 2023 for myastheniform syndrome.

**Fig 2:**
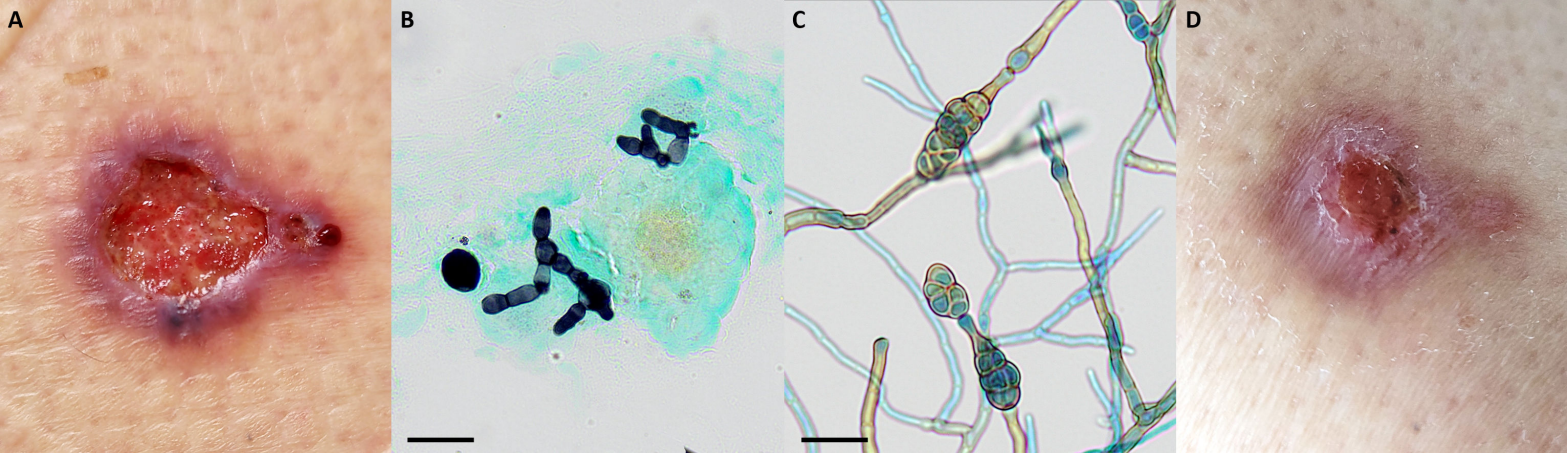
Cutaneous ulceration on the right thigh at the diagnosis (**A**). Grocott-Gomori’s methenamine silver staining of the cutaneous biopsy showing numerous fungal elements with branched and septate hyphae of approximately 5 µm in diameter, and budding cells reaching up to 15 µm in diameter, 500× magnification, scale bar 20 µm (**B**). Lactophenol-cotton blue mount of Sabouraud’s culture showing dictyospores 20–30 µm long and 9–18 µm wide, with a transverse septum at maturity and an apical conidiogenous cell with a beak shape, 500× magnification, scale bar 20 µm (**C**). Cutaneous ulceration on the right thigh after 1 month of treatment by posaconazole (**D**).

On admission, the patient reported having consulted his dermatologist 10 months ago, following the appearance of a punctiform nodule on the right thigh, without fever. At this stage, no exploration was performed, as the lesion was probably associated with actinic keratosis. Then, a progressive extension of the lesion was observed, reaching a diameter of 10 × 11 mm^2^ at the time of admission in infectious diseases department, associated with ulceration and necrosis (Fig. 2A). Blood analyses revealed slightly elevated C-reactive protein of 12.3 mg/L (<10.0 mg/L) and a chronic lymphopenia (<1.0 G/L) without associated neutropenia. A thoraco-abdomino-pelvic CT scan was performed but did not show secondary lesions.

A cutaneous biopsy was performed for microbiological and histological investigations. Histological analyses showed an inflammatory infiltrate of the dermis, with histiocytes, neutrophils, and giant cells accompanied by thick and branched hyphae of irregular caliber. Mycological investigations using Grocott-Gomori’s methenamine silver (GMS) stains for direct examination revealed numerous fungal elements with branched and septate hyphae of approximately 5 µm in diameter, and budding cells reaching up to 15 µm in diameter (Fig. 2B). After 8 days of culture, thick and woolly gray colonies grew on Sabouraud media at 35°C. Microscopic examination (lactophenol cotton blue mounting) enabled the identification of melanized multicellular conidia, arranged in branched chains and presenting a differentiated beak. Fungal growth revealed pear-shaped dictyospores (i.e., spores with longitudinal and transverse septates, Fig. 2C), specific to *Alternaria* genus. A Sanger sequencing (BigDye Terminator, Applied Biosystems) of the Internal Transcribed Spacer (ITS) identified with 100% of similarity an *Alternaria alternata* species complex (GenBank reference sequence accession number MH863109). The diagnosis was cutaneous alternariosis associated with concomitant acalabrutinib and corticosteroid treatment.

## DISCUSSION

*Alternaria* spp. are dematiaceous (melanized) molds with worldwide distribution, saprophytes of soil, air, or agents of decay and plant pathogens. Macroscopic colonies are gray to black with cottony growth after several days at 35°C on standard Sabouraud media (in our case 7 days). Microscopically, *Alternaria* spp. are characterized by conidial chains, pear-shaped dictyospores, characteristics which are useful for distinction among other molds that cause phaeohyphomycosis. Dictyospores produced by *Alternaria* species are 20–30 µm long and 9–18 µm wide, with a transverse septum at maturity, and have an apical conidiogenous cell with a beak shape. The acropetal spore production mode gives rise to chains of dictyospores (1). However, dictyospores can take several additional days to appear (in our case 10 days) and do not allow identification. Species-level identification is not required for treatment management, but is recommended for accurate documentation, especially when morphological identification may be complicated by limited sporulation of the causative pathogens (2). Identification can be achieved by matrix-assisted laser desorption ionization-time of flight mass spectrometry (MALDI-TOF MS), at least to the species complex level if the database allows reliable identification. To date, the two commercial MALDI-TOF databases, MBT IVD reference library (Brucker) and VITEK MS KB v3.2 (Biomerieux), include only *A. alternata*. However, the latest version of the Mass Spectrometry Identification database (MSI-2) includes more *Alternaria* species up to the species level. Molecular method based on the analysis of rDNA ITS sequence data remains the most accurate method for identifying clinically relevant *Alternaria* species that are morphologically similar (3).

*Alternaria* spp. had been frequently associated with hypersensitivity diseases such as hypersensitivity pneumonitis, bronchial asthma, and allergic sinusitis. Many other different types of human infections have also been reported, such as ocular infections, onychomycosis, cutaneous infections or, less frequently, disseminated disease in case of severe immunosuppression (3–5). The most common site of infection by *Alternaria* spp. is the skin, especially after skin trauma (1). The classic clinical presentation was erythema and desquamation or red papules progressing to ulceration of a unilocular plaque, especially with corticosteroid treatment. Complication of the disease towards a multilocular form was generally associated with disseminated alternariosis (6). *Alternaria* spp. are one of the most common causative agents of cutaneous phaeohyphomycosis in immunocompromised patients (2). Patients with hematological malignancies and solid organ transplant recipients are the most at risk of phaeohyphomycosis (7, 8).

*In vitro* antifungal susceptibility studies have shown that terbinafine, caspofungin, and azoles, such as itraconazole, voriconazole, or posaconazole, with the exception of fluconazole, have good activity against *Alternaria* spp. (9–11). Amphotericin B showed variable *in vitro* activity with a Minimum Inhibitory Concentration inhibiting the growth of 50% of the isolates (MIC_50_) of 0.25 µg/mL (9, 12). Flucytosine appears to have no *in vitro* activity on *Alternaria* spp. (13).

Although there are no recommendations for the treatment of *Alternaria* cutaneous infection, immunosuppression could be managed, but surgical excision of infected tissue along with antifungal therapy should be considered first (1, 2). The patient did not undergo surgical excision due to the limited area of the lesion. However, excision of the cutaneous lesion promotes clinical remission when immunosuppressive therapy adjustment is not possible and shortens the duration of treatment, which can last several months (1, 2). Itraconazole, and more generally azole antifungal agents other than fluconazole, are the most commonly reported treatments (8). Otherwise, a recent case of azole *in vivo* resistance has been reported for a strain of *A. alternata* with an itraconazole MIC of 2 mg/L (14). Nevertheless, interpretation of MIC values for *Alternaria* spp. is tricky in the absence of clinical breakpoints or epidemiologic cutoff values. Usually *Alternaria* spp. grow in subculture, but in our case, antifungal susceptibility testing was not available due to the lack of growth on RPMI medium, although the patient was not receiving antifungal prophylaxis. So, based on literature data and drug-drug interactions, the patient was treated with posaconazole (300 mg per day). After 1 month, the lesion had successfully improved (Fig. 2D). Four months after stopping antifungal treatment, the patient did not relapse.

This case highlights the value of mycological investigation of chronic skin involvement during IL-2-inducible tyrosine kinase (ITK) inhibitor therapy. Acalabrutinib is a second-generation covalent Bruton’s tyrosine kinase inhibitor (BTKi) for the treatment of B-cell malignancies, with fewer off-target effects and a 41% reduced risk of treatment discontinuation compared to ibrutinib (15). Safety data from phases 1, 2, and 3 studies reported a fungal infection rate of 9.0% (63 out of 693) among all documented infections. More precisely, a serious fungal infection rate was reported for 11 patients, including three cases of aspergillosis (two were fatal), one case of disseminated cryptococcosis and one fatal case of candidemia (16). In another phase 3 trial, opportunistic fungal infections occurred in 3.8% of acalabrutinib patients, manifesting in five cases of aspergillosis and five cases of pneumocystosis (17). More recently, five additional cases of invasive pulmonary aspergillosis and one case of concomitant central nervous system infections with *Cryptococcus neoformans* and *Aspergillus fumigatus* were reported in patients treated with acalabrutinib (18, 19). Another case of invasive nervous system infection in patients receiving long-term acalabrutinib treatment has been reported, but the fungal strain, *Aspergillus*-like, has not been investigated (20). Here, we report the first case of *Alternaria* cutaneous infection associated with acalabrutinib treatment. It was suggested an altered neutrophils response against conidial germination of *Aspergillus* and decreased reactive oxygen species production during acalabrutinib treatment (18). In the present case, it cannot be excluded that corticosteroid treatment for myastheniform syndrome may also have an impact on the occurrence of invasive fungal infections, as previously reported for ibrutinib (21).

In conclusion, clinicians should be aware that invasive mold infection associated with concomitant acalabrutinib and corticosteroid treatment may also involve phaeohyphomycosis. In this circumstance, any chronic ulcero-necrotic skin disease in immunocompromised patients should be investigated with mycological examination to prevent the risk of disseminated invasive fungal infection.

## SELF-ASSESSMENT QUESTIONS

1. Which of the following statements about acalabrutinib is true?
  - It is a first-generation Bruton’s tyrosine kinase inhibitor.
  - It is indicated in T-cell line malignancies.
  - It is indicated in B-cell line malignancies.
  - It is indicated in the treatment of acute leukemia.
2. Which of the following laboratory methods is not appropriate for diagnosing *Alternaria*-type cutaneous mycosis?
  - Serology
  - Histopathologic examination of cutaneous biopsy
  - Mycological culture of cutaneous biopsy
  - Fungal identification by ITS sequencing of the strain from culture
3. Which antifungal agent is commonly used to treat cutaneous *Alternaria* species infection?
  - Flucytosine
  - Fluconazole
  - Itraconazole
  - Amphotericin B

## ANSWER TO SELF-ASSESSMENT QUESTIONS

1. Which of the following statements about acalabrutinib is true?
  - It is a first-generation Bruton’s tyrosine kinase inhibitor.
  - It is indicated in T-cell line malignancies.
  - It is indicated in B-cell line malignancies.
  - It is indicated in the treatment of acute leukemia.

Answer: c. Acalabrutinib was developed after ibrutinib in the treatment of CLL, a malignant disease of the B cell line. Acalabrutinib is more selective than ibrutinib and improves the tolerability of prolonged treatment with fewer interruptions.

- Which of the following laboratory methods is not appropriate for diagnosing *Alternaria*-type cutaneous mycosis?
  - Serology
  - Histopathologic examination of cutaneous biopsy
  - Mycological culture of cutaneous biopsy
  - Fungal identification by ITS sequencing of the strain in culture

Answer: a. Cutaneous biopsy is performed to document the infection. Histopathologic examination is specific but not sensitive. In addition to standard microbiological investigations, mycological analysis allows inoculation of specific media with appropriate growth delay (several days to weeks for *Alternaria* spp.). Sequencing the ITS region can distinguish genus and species when microscopic analysis is non-contributory.

- Which antifungal agent is commonly used to treat cutaneous *Alternaria* species infection
  - Flucytosine
  - Fluconazole
  - Itraconazole
  - Amphotericin B

Answer: c. Flucytosine and fluconazole have shown no *in vitro* activity against *Alternaria* spp. Amphotericin B has shown variable *in vitro* activity and was not commonly used to treat *Alternaria* species cutaneous infections. Itraconazole and Posaconazole have shown good *in vitro* activity. Itraconazole is the treatment most frequently reported in clinical cases Posaconazole has fewer drug interactions than itraconazole.

**TAKE-HOME POINTS**

- Acalabrutinib is a new line of treatment for CCL with fewer off-target effects than ibrutinib and will be increasingly used in the coming years.
- The risk of invasive fungal infection in patients with chronic skin lesions receiving BTKi concomitantly with corticosteroid therapy should not be overlooked.
- Cutaneous alternariosis is a typical example of an infection that can occur in immunocompromised patients.
- Species identification to guide antifungal treatment and surgical excision, combined with management of the patient's immunosuppression, is key to therapeutic success.
